# Comparative Analysis of Commercial and Home-Made Media on RSPO1/S6R Axis in Organoids with Different Wnt Backgrounds: A Methodological Guide for the Selection of Intestinal Patient-Derived Organoids Culture Media

**DOI:** 10.3390/ijms252111526

**Published:** 2024-10-26

**Authors:** Giulia Calafato, Chiara Alquati, Alice Bernardi, Floriana Jessica Di Paola, Luigi Ricciardiello

**Affiliations:** 1 IRCCS Azienda Ospedaliero-Universitaria di Bologna, 40138 Bologna, Italy; giulia.calafato@aosp.bo.it (G.C.);; 2 Department of Medical and Surgical Sciences, University of Bologna, 40138 Bologna, Italy; chiara.alquati2@unibo.it (C.A.);; 3 Centre for Applied Biomedical Research (CRBA), University of Bologna, 40138 Bologna, Italy

**Keywords:** colorectal cancer (CRC), intestinal patient-derived organoid (PDO), organoids culture media, phospho-S6 ribosomal protein (p-S6R), R-spondin 1 (RSPO1)

## Abstract

WNT3A is an intestinal ligand triggering the Wnt/β-catenin (Wnt) pathway, which can be enhanced by R-spondin 1 (RSPO1) through the RSPO1–LGR axis or antagonized by the adenomatous polyposis coli (APC) protein supporting β-catenin-degradation. Wnt interplays with several pathways including PI3K/mTOR (mTOR). In this study, we evaluated the influence of WNT3A-commercial and home-made culture media and RSPO1 protein on the Wnt and mTOR interplay in non-APC and APC-mutated intestinal patient-derived organoids (PDOs). Normal mucosa (NM) of sporadic CRC and FAP PDOs were cultured with: WNT3A-lacking/containing commercial (A/A+B) or home-made (BASAL/WNT3A-conditioned medium (CM)±RSPO1) media. In non-APC-mutated-PDOs (CRC-NM), WNT3A-CM, over commercial A+B, strongly activated Wnt-target-genes *CCND1* and *c-MYC*. Most importantly, the addition of RSPO1 to home-made WNT3A-CM or A+B led to the downregulation of the mTOR-downstream-effector phospho-S6 ribosomal protein (p-S6R), highlighting the activation of the RSPO1–pS6R in both non-APC (CRC-NM) and APC-mutated (FAP-NM) PDOs, independently from *LGR5* gene expression modulation. Our work demonstrates that home-made WNT3A-CM strongly impacts the crosstalk between Wnt and mTOR over commercial media, and proposes RSPO1 as a key regulator of the RSPO1–p-S6R axis in both non-APC and APC-mutated PDOs. Together, these findings represent an important methodological guide for scientists working in these fields to select the most appropriate intestinal PDO media.

## 1. Introduction

WNT3A is one of the main extracellular ligands triggering the canonical Wnt/β-catenin (Wnt) pathway, leading to the β-catenin-induced transcription of Wnt target genes [1]. Among them, the Wnt-target proliferative stem cell marker *LGR5* gene encodes for the receptor of R-spondin 1 (RSPO1), a Wnt agonist involved in the positive regulation of signaling, acting on the Frizzled/Lrp receptor complex [2]. Conversely, as part of the β-catenin destruction complex, the adenomatous polyposis coli (APC) is directly involved in the negative regulation of the Wnt pathway through the modulation of GSK3β/CK1-dependent β-catenin phosphorylation, addressing it to the proteasomal degradation [3]. To note, in APC-mutated settings, the destruction complex is compromised and leads to the accumulation of β-catenin, which drives the uncontrolled transcription of Wnt target genes (*AXIN2*, *CCND1*, *c-MYC*, and *LGR5*), thus causing Wnt dysregulation [4,5,6]. Accordingly, somatic APC mutations represent an early event occurring in 85% of sporadic colorectal cancer (CRC), while germline mutations are responsible for the CRC-predisposing syndrome familial adenomatous polyposis (FAP).

To date, the interplay of Wnt/β-catenin signaling with several pathways implicated in CRC carcinogenesis is well-known, including the phosphatidylinositol-3-kinase/mammalian target of rapamycin (PI3K/mTOR) [7]. The Wnt and PI3K/mTOR (mTOR) pathways are both critically involved in CRC onset and progression, and regulate each other through several feedback mechanisms that drive target-therapy resistance [8]. In the last few years, the use of 3D intestinal patient-derived organoids (PDOs) has represented an important step forward to study the molecular mechanisms underlying CRC carcinogenesis, particularly in uncovering the dual role of Wnt signaling, which in turn regulates gut homeostasis or triggers CRC onset [9]. Extensive efforts have been made to optimize long-term culture media formulations for human intestinal PDOs, aiming to recreate the original stromal-derived niche and maintaining the balance between proliferative (Wnt) and differentiation (BMP) signals [10]. Accordingly, WNT3A (recombinant or conditioned medium-CM), EGF, BMP-inhibitor noggin, and Wnt signal amplifier R-spondin 1 (RSPO1) are the primary components of media used for the long-term culture of human intestinal colon organoids together with A8301 (ALK-5 inhibitor), SB202190 (p38 MAPK inhibitor), and Y-27632 (ROCK inhibitor), while several antioxidants, hormones, and vitamins (N2, B27, N-acetylcysteine, gastrin, nicotinamide) are important additional variables [11]. In this field, several studies have proposed specific formulations to allow for the long-term culture, expansion, or differentiation of intestinal organoids, depending also on the genetic features of the original tissue/biopsy [11,12,13,14]. In the past years, different compositions of media have been studied and described, mainly focusing on their influence on organoid drug-response or stemness maintenance [15,16,17]. However, it remains unclear as to how different home-made and commercial ready-to-use formulations of intestinal organoid culture media could influence the levels of interaction between Wnt/β-catenin and PI3K/mTOR. Moreover, it has not been clarified whether the presence of an APC-mutated setting could be an additional variable that modulates the crosstalk between the two pathways under different media compositions. Based on these considerations, the aim of the present study was to evaluate the differential impact of WNT3A in commercial or home-made media in the crosstalk between the Wnt and mTOR pathway activation in APC and non-APC mutated intestinal PDOs, also delving deeper into the role of the Wnt-amplifier RSPO1.

## 2. Results and Discussion

### 2.1. Commercial Wnt Supplement and Home-Made WNT3A-CM Induce Comparable Wnt Activation in HEK293 STF Reporter Cells

**Figure 1:**
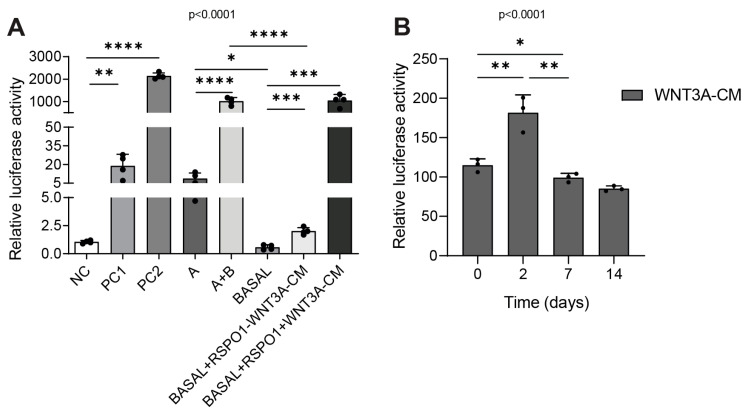
Canonical Wnt/β-catenin pathway activation in the HEK 293 STF cell reporter cell line in commercial and home-made-cultured non-APC mutated CRC NM PDOs. (**A**) Luciferase assay of HEK 293 STF cells cultured for 24 h with different intestinal organoid culture media (n = 4). (**B**) Luciferase-based quality control test of WNT3A-CM in HEK 293 STF cells cultured for 24 h with freshly thawed medium (termed 0) or 2/7/14-days-thawed medium (n = 3). Experiments were independently repeated at least three times and the values are reported as the mean ± SD. Data were analyzed with the parametric Student’s *t*-test according to Shapiro–Wilk normality test results. * *p* < 0.05, ** *p*  < 0.01, *** *p*  < 0.001, **** *p* < 0.0001. NC = negative control; PC1 = positive control 1; PC2 = positive control 2; A = WNT3A-lacking commercial medium composed of DMEMF12 and Component A in a 1:1 ratio; A+B = WNT3A-containing commercial medium composed of Component A and Supplement B in a 1:1 ratio (exact formulation relative to other factors present is not known); BASAL = WNT3A-lacking home-made basal medium; WNT3A-CM = WNT3A-containing complete home-made medium, with or without R-spondin1 (+/−RSPO1).

To assess whether the presence of WNT3A ligands in commercial or home-made media could activate the canonical Wnt pathway within the same range of intensity, we used HEK 293 STF cells as the luciferase reporter cell line. Cells were cultured for 24 h with the following commercial and home-made media: WNT3A lacking/containing commercial (A/A+B) and home-made medium (BASAL), supplemented with R-spondin1 (RSPO1), and with or without WNT3A conditioned medium (+/−WNT3A-CM). NC was used as the negative control while PC1 and PC2 were the positive controls. The detailed media composition is reported in Table S1. After 24 h, we measured the luciferase activity, reflecting the activation of canonical Wnt signaling. The results indicated that compared to the corresponding WNT3A-lacking media (A and BASAL), A+B and BASAL+RSPO1+WNT3A-CM induced a similar activation of Wnt signaling (*p* < 0.0001 and *p* = 0.0003, respectively), which was closer to the value of the PC2 positive control (Figure 1A). Moreover, the addition of RSPO1 to the BASAL medium alone induced a significant enhancement of Wnt signaling (*p* = 0.0003), although with lower levels compared to both PC1 or PC2 (Figure 1A). Since RSPO1 is a Wnt agonist responsible for the enhancement of the Wnt receptors’ stabilization on the cell surface, this result was aligned with the common literature stating that RSPO1 is sufficient for the amplification of the Wnt pathway but requires the presence of canonical Wnt ligands to trigger the signaling more efficiently, even in the PDO-media formulations [18]. Compared to the BASAL medium, we detected a residual Wnt activity in commercial A medium (*p* = 0.01), suggesting the presence of other factors in this formulation that slightly sustain Wnt pathway activation as Wnt-amplifier proteins (Figure 1A). In addition, we tested the stability of the home-made WNT3A-CM (without BASAL) over time and its ability to induce Wnt. Thus, we cultured HEK 293 STF cells with WNT3A-CM (without BASAL) freshly thawed (named time 0) or thawed by 2, 7, and 14 days, and after 24 h, and we tested the luciferase activity. NC and PC1/2 were used as the negative and positive controls, respectively. Our analysis showed a significant reduction in Wnt activation when the cells were cultured with the 7-days-thawed batch (compared to the 2-days-thawed batch—*p* = 0.0036) (Figure 1B). An overview of the quality test results over time including WNT3A-CM, negative, and positive controls is reported in Figure S1A. According to the common literature, one of the limits of PDO cultures is that cells located in the center of organoids undergo decreased nutrition supply during organoid growth, subsequently resulting in cell death [19]. In addition, according to our experience, a restricted time point of 48 h avoids an overgrowth of human non-cancerous organoids. The results of the WNT3A-CM stability test, associated with the evidence reported in the literature characterizing 3D culture models, allowed us to choose 48 h as the organoids’ culture time point for the subsequent set of experiments.

### 2.2. WNT3A-Containing Commercial and Home-Made Media Influence the Activation of Wnt-β-Catenin Differently in Non-APC Mutated CRC NM PDOs

**Figure 2:**
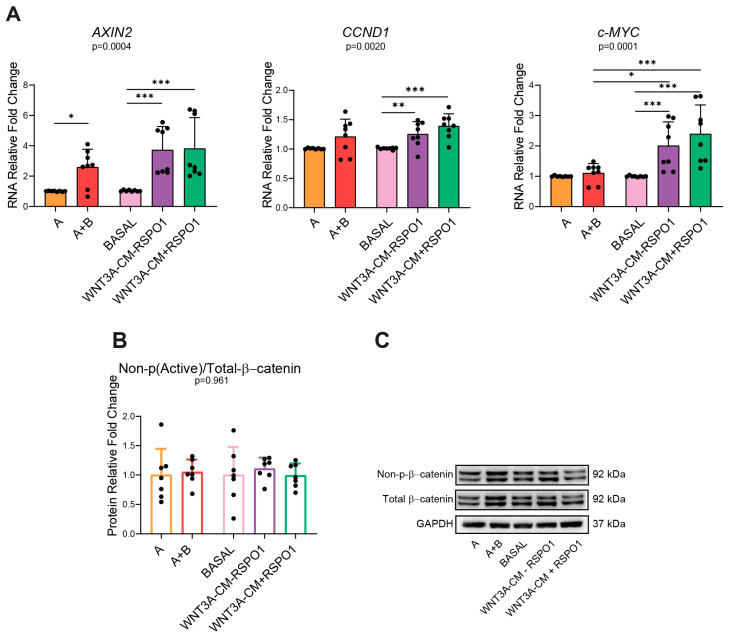
Different activation of Wnt/β-catenin in commercial and home-made-cultured non-APC mutated CRC NM PDOs. (**A**) RNA expression of Wnt/β-catenin target genes *AXIN2*, *CCND1*, and *c-MYC* of CRC NM PDOs cultured for 48 h with the indicated media (n = 8, of which PT10 = 6, PT13 = 1, PT16 = 1). (**B**) WB analysis of non-p(Active)/total β-catenin protein ratio in CRC NM PDOs cultured for 48 h with the specified media as a marker of Wnt activation (n = 7, of which PT10 = 5, PT13 = 1, PT16 = 1). (**C**) Representative WB imaging of non-p(Active)-β-catenin, total β-catenin, p-S6R, and GAPDH (n = 7); GAPDH was used as the loading control (cropped blots). Experiments were independently repeated at least three times and the values are reported as mean ± SD. Data were analyzed with the parametric Student’s *t*-test (*CCND1* and non-p(Active)/total β-catenin) and non-parametric Mann–Whitney test (*AXIN2* and *c-MYC*), according to the Shapiro–Wilk normality test results. * *p* < 0.05, ** *p*  < 0.01 and *** *p*  < 0.001. A = WNT3A-lacking commercial medium composed of DMEMF12 and Component A in a 1:1 ratio; A+B = WNT3A-containing commercial medium composed of Component A and Supplement B in a 1:1 ratio (exact formulation relative to other factors present is not known); BASAL = WNT3A-lacking home-made basal medium; WNT3A-CM = WNT3A-containing complete home-made medium, with or without R-spondin1 (+/−RSPO1).

Once assessed that B supplement and WNT3A-CM activated Wnt signaling within a similar range, we decided to further investigate in more depth as to whether the commercial and home-made media could differently activate the Wnt target genes and the related effector proteins. Since activation of the Wnt pathway is influenced by the mutational setting of the tissue itself [20], we first decided to use the non-APC mutated CRC NM PDOs to exclude any possible genetic-background-derived interference and exclusively investigate the molecular changes given by the different types of media used. Moreover, since RSPO1 is known to enhance the Wnt pathway through the RSPO1–LGR4/5 axis [21] and is usually included in most of the optimized long-term culture media formulations [11], we added RSPO1 to the WNT3A-CM medium to understand its potential additional contribution in sustaining Wnt signaling in the home-made medium compared to the commercial A+B, whose formulation should already include all the known-intestinal niche factors. Wnt target genes and proteins were evaluated through RT-qPCR and WB in CRC NM PDOs cultured for 48 h with different types of growth media: WNT3A-lacking/containing commercial (A/A+B) and WNT3A-CM lacking/containing home-made media (BASAL/WNT3A-CM) with or without RSPO1 (WNT3A-CM+/−RSPO1). As expected, the analysis performed on CRC NM PDOs indicated that all the media containing WNT3A (A+B and WNT3A-CM-RSPO1) significantly increased *AXIN2* gene expression within a similar range compared to the corresponding WNT3A-lacking media (*p* = 0.0104 and *p* = 0.0007, respectively) (Figure 2A). Surprisingly, compared to its corresponding media without WNT3A (BASAL), WNT3A-CM-RSPO1 significantly enhanced the expression of the *CCND1* (*p* = 0.0051) and *c-MYC* (*p* = 0.0007) genes, while A+B only showed a positive trend compared to A (*p* = 0.0689 and *p* = 0.1049, respectively) (Figure 2A). Interestingly, the addition of RSPO1 to WNT3A-CM did not significantly increase the *AXIN2, CCND1*, and *c-MYC* gene expression, and the two media showed similar expression levels of these markers (Figure 2A). This result could be related to the fact that, among the four RSPO proteins (RSPO1-4), RSPO1 was the least potent agonist of the canonical Wnt [22]. Thus, our results revealed that, independently from the addition of RSPO1, the home-made WNT3A-CM media activated the Wnt pathway more strongly than A+B, making the WNT3A-containing home-made media more prone to modulating the Wnt-proliferation-target genes *CCND1* and *c-MYC*. To further consolidate this result, we investigated whether commercial or home-made media triggered the accumulation of active β-catenin differently. Unexpectedly, despite the pronounced activation status of the Wnt target genes (*CCND1* and *c-MYC*) in the home-made WNT3A-CM media, WB analysis showed similar levels between the commercial and home-made media in the expression of non-phospho (Active)/total-β-catenin (Figure 2B,C). In addition, no significant differences were detected between media containing or lacking WNT3A (Figure 2B,C). Accordingly, it has been reported that the cadherin-free form of β-catenin unphosphorylated at S37 and T41, typically referred to as transcriptionally active β-catenin (ABC), is a minor nuclear-enriched monomeric form of β-catenin, implying that β-catenin transcriptional activity can also occur in the absence of its robust nuclear accumulation [23]. Our data could also be possibly explained according to the distributive model proposed for the destruction complex where, upon Wnt stimulation, some destruction complexes maintain their activity and relocalize to the cellular membrane [24]. In addition, we also hypothesize that this result could be due to the optimization strategies used for the formulation of commercial and home-made media, which tend to maintain a more homeostatic-like activation of the Wnt pathway for the long-term culture of intestinal organoids.

### 2.3. RSPO1 in the Home-Made WNT3A-CM Medium Guides LGR5-Dependent p-S6R Downregulation in Non-APC Mutated CRC NM PDOs

**Figure 3:**
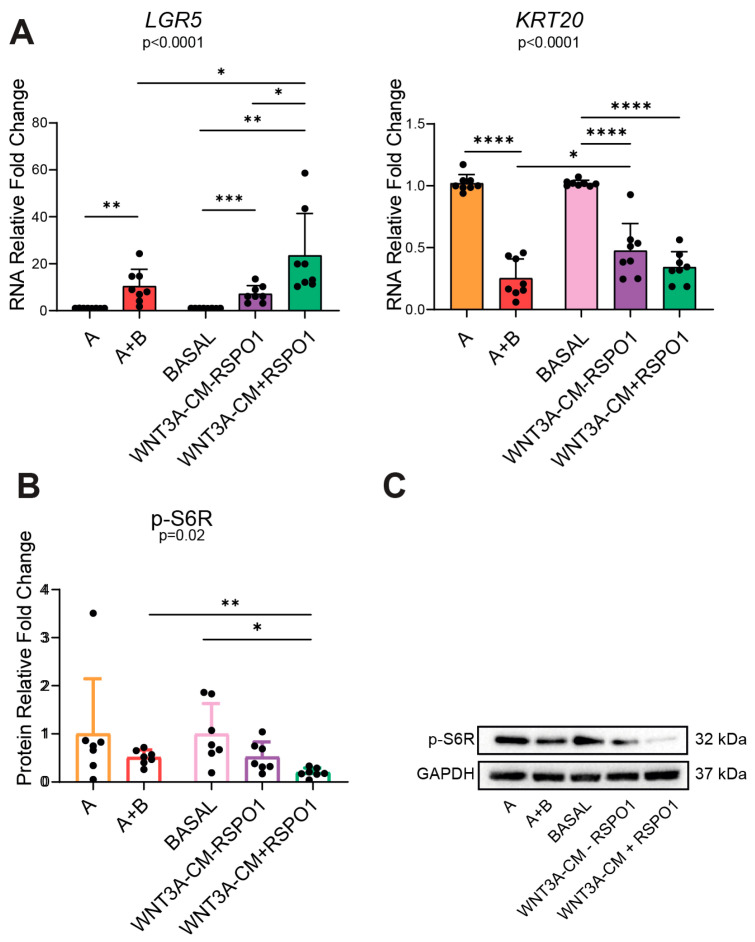
Different activation of intestinal stemness/differentiation markers and PI3K/mTOR in commercial and home-made-cultured non-APC mutated CRC NM PDOs. (**A**) RNA expression of stemness (*LGR5*) and differentiation (*KRT20*) intestinal markers in 48 h-cultured CRC NM PDOs (n = 8, of which PT10 = 6, PT13 = 1, PT16 = 1). (**B**) WB analysis of mTOR effector p-S6R protein in CRC NM PDOs cultured for 48 h with the indicated media (n = 7, of which PT10 = 5, PT13 = 1, PT16 = 1). (**C**) Representative WB imaging of non-p(Active)-β-catenin, total β-catenin, p-S6R, and GAPDH (n = 7); GAPDH was used as the loading control (cropped blots). Experiments were independently repeated at least three times and the values are reported as mean ± SD. Data were analyzed with the parametric Student’s *t*-test (*LGR5* and *KRT20*) and non-parametric Mann–Whitney test (p-S6R), according to the Shapiro–Wilk normality test results. * *p* < 0.05, ** *p*  < 0.01, *** *p*  < 0.001, **** *p* < 0.0001. A = WNT3A-lacking commercial medium composed of DMEMF12 and Component A in a 1:1 ratio; A+B = WNT3A-containing commercial medium composed of Component A and Supplement B in a 1:1 ratio (exact formulation relative to other factors present is not known); BASAL = WNT3A-lacking home-made basal medium; WNT3A-CM = WNT3A-containing complete home-made medium, with or without R-spondin1 (+/−RSPO1).

On the basis of the previous results and considering that the Wnt pathway is involved in the homeostasis of adult intestinal stem cells (ASCs) [25], we decided to investigate whether the use of commercial or home-made media also triggered a different activation of *LGR5*, a Wnt-target gene and intestinal stem cells marker, and of *KRT20*, an intestinal differentiation marker, in CRC NM PDOs. As expected, the RT-qPCR analysis indicated that compared to the corresponding reference media, WNT3A-containing media A+B and WNT3A-CM significantly enhanced intestinal stemness (*LGR5*) (*p* = 0.0024 and *p* = 0.0002, respectively) and reduced differentiation (*KRT20*) (*p* < 0.0001 and *p* < 0.0001, respectively) (Figure 3A). Moreover, the addition of RSPO1 to WNT3A-CM significantly doubled the expression of the *LGR5* gene compared to A+B (*p* = 0.0169) and WNT3A-CM-RSPO1 (*p* = 0.0347), which in contrast, presented similar *LGR5* transcriptional levels (Figure 3A). Since we found a lower expression of *LGR5* in the commercial A+B media compared to WNT3A-CM+RSPO1, and RSPO1 is associated with the well-characterized boost-regulation axis with LGR5 proteins to enhance Wnt signaling [2], our data suggested allegedly lower levels of RSPO1 in the commercial A+B. This result was also in line with the functionally non-equivalent but cooperative interaction proposed for Wnt and RSPO ligands, in which it was hypothesized that Wnt ligands confer basal competence by maintaining LGR5 expression, which allows RSPO to further increase it and actively drive and specify the self-renewal of Lgr5+ ISCs [26].

It is well-recognized that the Wnt/β-catenin and the PI3K/mTOR pathways are finely interrelated through multiple mechanisms [8,27]. Therefore, we decided to investigate how the different modulation of the Wnt pathway induced by WNT3A and RSPO1 in commercial or home-made media could influence the activation of PI3K/mTOR. For this reason, we analyzed phospho-S6 ribosomal protein (p-S6R) expression as a PI3K/mTOR downstream effector. Interestingly, WB analysis showed that compared to the corresponding media without WNT3A, A+B (*p* = 0.2086) and WNT3A-CM-RSPO1 (*p* = 0.1375) induced a trend of downregulation for p-S6R (Figure 3B,C). Surprisingly, the addition of RSPO1 to WNT3A-CM significantly suppressed p-S6R protein expression (*p* = 0.0101 compared to BASAL; *p* = 0.0051 compared to A+B), thus showing a stronger downregulation of the protein compared to the one observed in A+B or WNT3A-CM-RSPO1 (Figure 3B,C). This suggests the existence of a direct regulation axis guided by RSPO1, which suppresses p-S6R protein expression by enhancing *LGR5* gene expression. The result could be associated with the recent proposed mechanism linking mTORC1 to intestinal stemness. Indeed, according to He D. et al., mTOR activation increased the protein synthesis of MKK6 and augmented activation of the p38 MAPK-p53 pathway, leading to decreases in the number and activity of intestinal stem cells as well as villus size and density [28]. However, another study performed on mouse models reported no substantial changes in LGR5 expression in the WT, mTOR^ΔIEC^, Rptor^ΔIEC^, and Rictor^ΔIEC^ ileal epithelium, suggesting an independent regulation between LGR5 and mTOR [29]. Together with the previous observations on the expression of the Wnt target genes, our results highlight that compared to WNT3A, RSPO1, as a growth factor in the intestinal organoid media, is involved in a stronger modulation of the negative feedback loop between the Wnt/β-catenin and PI3K/mTOR pathways, directly acting on p-S6R.

### 2.4. Different Activation of Wnt/β-Catenin and PI3K/mTOR Triggered by WNT3A-Containing Commercial and Home-Made Media Did Not Affect Organoid Viability

**Figure 4:**
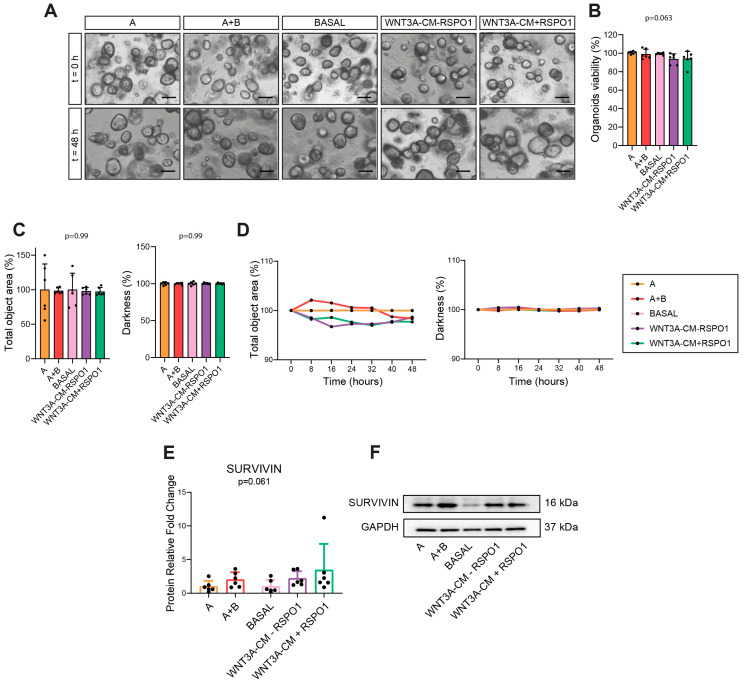
Effect of commercial and home-made media on the viability and morphology of non-APC-mutated CRC NM PDOs. (**A**) Representative images of CRC NM PDOs cultured with the indicated media at 48 h (40× magnification, scale bar = 100 µm) (n = 6); representative pictures were captured through an EVOS M5000 Microscope. (**B**) Analysis of CRC NM PDO viability cultured for 48 h with the different culture media (n = 6, of which PT10 = 4, PT13 = 1, PT16 = 1). (**C**) Morphological analysis of the total object area and darkness of CRC NM PDOs in different culture media at the 48 h time point (n = 6, of which PT10 = 4, PT13 = 1, PT16 = 1). (**D**) Time-lapse morphological analysis of cultured-CRC NM PDOs. Morphology (total object area and darkness) was monitored every 8 h for 48 h (n = 6, of which PT10 = 4, PT13 = 1, PT16 = 1). (**E**,**F**) WB analysis and representative imaging of SURVIVIN protein in CRC NM PDOs cultured for 48 h with the specified media (n = 6, of which PT10 = 4, PT13 = 1, PT16 = 1); GAPDH was used as the loading control (cropped blots). Experiments were independently repeated six times and values are shown as the mean ± SD. Data were analyzed with the parametric Student’s *t*-test (viability assay and morphological analysis) and non-parametric Mann–Whitney test (SURVIVIN), according to the Shapiro–Wilk normality test results. A = WNT3A-lacking commercial medium composed of DMEMF12 and Component A in a 1:1 ratio; A+B = WNT3A-containing commercial medium composed of Component A and Supplement B in a 1:1 ratio (exact formulation relative to other factors present is not known); BASAL = WNT3A-lacking home-made basal medium; WNT3A-CM = WNT3A-containing complete home-made medium, with or without R-spondin1 (+/−RSPO1).

We observed that within a 48-h time frame, WNT3A-lacking/containing media provoked a different modulation of the Wnt and mTOR pathways and intestinal stemness. Therefore, we wondered whether organoid viability could be affected by the different culture media conditions. Surprisingly, we found no differences in organoid viability and morphology in CRC NM PDOs cultured for 48 h either with WNT3A-lacking/containing commercial or home-made media (Figure 4A–D). In addition, the time-lapse imaging and morphological analysis (total area, object count and darkness) performed on CRC NM PDOs every 8 h for 88 h excluded any possible short-term or long-term morphological changes to the commercial or home-made-cultured-organoids (Figures S2 and S3). Since the morphological parameters chosen are important factors to indirectly assess proliferation or viability, these results support the interchangeability of commercial or home-made media above all in the drug screening assay. We further confirmed this evidence by evaluating SURVIVIN protein expression, a protein involved in apoptosis inhibition and cell cycle regulation [30]. Indeed, regarding the organoid viability, we did not find relevant alterations, even if a positive trend was observed between all WNT3A-containing media compared to those without WNT3A (Figure 4E,F). It has previously been reported that 4-days removal of WNT3A induced a shift toward the differentiative over the proliferative behavior of the intestinal epithelium [15]. However, we showed that in short-term (48 h) viability experiments, the use of a commercial or home-made media and the addition of WNT3A or RSPO1 was completely irrelevant to the organoid viability. Our results further indicate that although the short-term organoids cultured with home-made and commercial media were sufficient to modulate the Wnt and PI3K/mTOR pathways differently, this was not reflected in any macroscopical change related to the organoid viability and morphology.

### 2.5. In FAP NM PDOs, A+B Induced a Lower LGR5 Activation Compared to CRC NM PDOs Without Affecting p-S6R Downregulation

**Figure 5:**
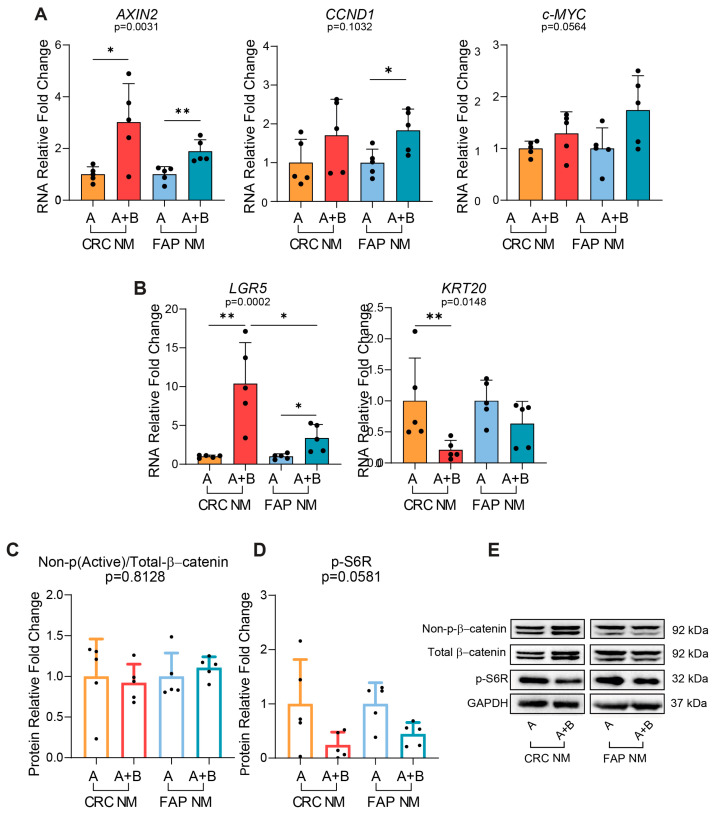
A+B-cultured-APC-mutated FAP NM PDOs induce lower LGR5 activation without affecting p-S6R downregulation. (**A**) RNA expression analysis of the Wnt/β-catenin target genes *AXIN2*, *CCND1*, and *c-MYC* in CRC NM and FAP NM PDOs cultured for 48 h in A and A+B (n = 5 of which, FAP NM-01 = 2, FAP NM-03 = 3, PT10 CRC NM = 3, PT13 CRC NM = 1, PT16 CRC NM = 1). (**B**) RNA expression analysis of stemness (*LGR5*) and differentiation (*KRT20*) intestinal markers in CRC NM and FAP NM PDOs cultured for 48 h in A and A+B (n = 5 of which, FAP NM-01 = 2, FAP NM-03 = 3, PT10 CRC NM = 3, PT13 CRC NM = 1, PT16 CRC NM = 1). (**C**,**D**) WB analysis of non-p(Active)/total β-catenin protein ratio and p-S6R in CRC NM and FAP NM PDOs cultured for 48 h with A and A+B (n = 5 of which, FAP NM-01 = 2, FAP NM-03 = 3, PT10 CRC NM = 3, PT13 CRC NM = 1, PT16 CRC NM = 1). (**E**) Representative WB imaging of non-p(Active)-β-catenin, total β-catenin, p-S6R, and GAPDH in CRC NM and FAP NM PDOs cultured for 48 h with A and A+B (n = 5 of which, FAP NM-01 = 2, FAP NM-03 = 3, PT10 CRC NM = 3, PT13 CRC NM = 1, PT16 CRC NM = 1); Representative WB imaging of FAP NM (A/A+B) and CRC NM (A/A+B) (cropped blots). GAPDH was used as the loading control. Experiments were independently repeated five times and values are shown as mean ± SD. Data were analyzed with the parametric Student’s *t*-test (*AXIN2*, *c-MYC*, *CCND1*, *LGR5*, non-p(Active)/total β-catenin and p-S6R) and non-parametric Mann–Whitney test (*KRT20*), according to the Shapiro–Wilk normality test results. * *p* < 0.05, ** *p*  < 0.01. A = WNT3A-lacking commercial medium composed of DMEMF12 and Component A in a 1:1 ratio; A+B = WNT3A-containing commercial medium composed of Component A and Supplement B in a 1:1 ratio (exact formulation relative to other factors present is not known).

To further explore whether the presence of an intrinsic status of Wnt activation could perturbate the Wnt and mTOR interplay ex vivo, we isolated FAP NM PDOs from the normal mucosa of FAP patients because they recapitulated an already Wnt-deranged system triggered by a single-hit germline mutation in the APC gene. To verify the mutational status of the APC gene in FAP NM biopsies, we extracted the genomic DNA from FAP01 and -03 NM and performed APC Sanger sequencing. As reported in Table S2, we found that FAP01 and -03 NM tissues presented an APC mutation. Moreover, to directly investigate the specific influence of the genetic mutational setting on the crosstalk between Wnt and mTOR, we decided to culture the APC-mutated FAP NM PDOs for 48 h only in A and A+B, since these media had a weaker effect on modulating the Wnt and mTOR pathways compared to the home-made media (BASAL and WNT3A-CM) in CRC NM PDOs. We found no significant differences in the gene expression of *AXIN2* (*p* = 0.1349), *CCND1* (*p* = 0.8012), *c-MYC* (*p* = 0.2332), and *KRT20* (*p* = 0.3571) (Figure 5A,B) as well as in the non-phospho (Active)/total-β-catenin protein expression (*p* = 0.1581) (Figure 5C,E) between FAPNM and CRC NM PDOs cultured in the A+B medium. Conversely, *LGR5* gene expression was the only Wnt-target gene to be affected and resulted in a lower activation in A+B-cultured FAP NM compared to CRC NM PDOs (*p* = 0.0229) (Figure 5B). More importantly, the statistically significant changes observed in *LGR5* activation between A+B-cultured FAP NM and CRC NM PDOs did not result in a substantial differential effect in the expression of the p-S6R protein, which was indeed similarly downregulated (*p* = 0.1879) (Figure 5D,E). Recently, King CM et al. described an alternative mechanism linking TCF7L1 to *LGR5* repression by binding to a consensus TCF binding element (TBE) within a novel Wnt-responsive DNA element (WRE) located at the *LGR5* proximal promoter in APC-mutated CRC cell lines. This mechanism could possibly explain the lower activation of the *LGR5* gene that we found in the APC-mutated setting compared to the wild-type one [31]. In addition, Okamoto and colleagues showed that protein kinase R-like endoplasmic reticulum kinase (PERK) is involved in LGR5 downregulation during endoplasmic reticulum stress in the APC-mutated HT29 cell lines [32]. Thus, our result revealed that the intrinsic activation status of Wnt, triggered by the presence or absence of an APC-mutated setting, distinguished the range of *LGR5* gene activation without affecting the levels of p-S6R downregulation. This suggests that the genetic background and *LGR5* itself can be excluded from the variables that guide the strength of the negative feedback acting on p-S6R in the APC-mutated FAP NM PDOs.

### 2.6. RSPO1 Exacerbates p-S6R Downregulation in APC-Mutated FAP NM PDOs Without Affecting the Levels of LGR5 Activation

**Figure 6:**
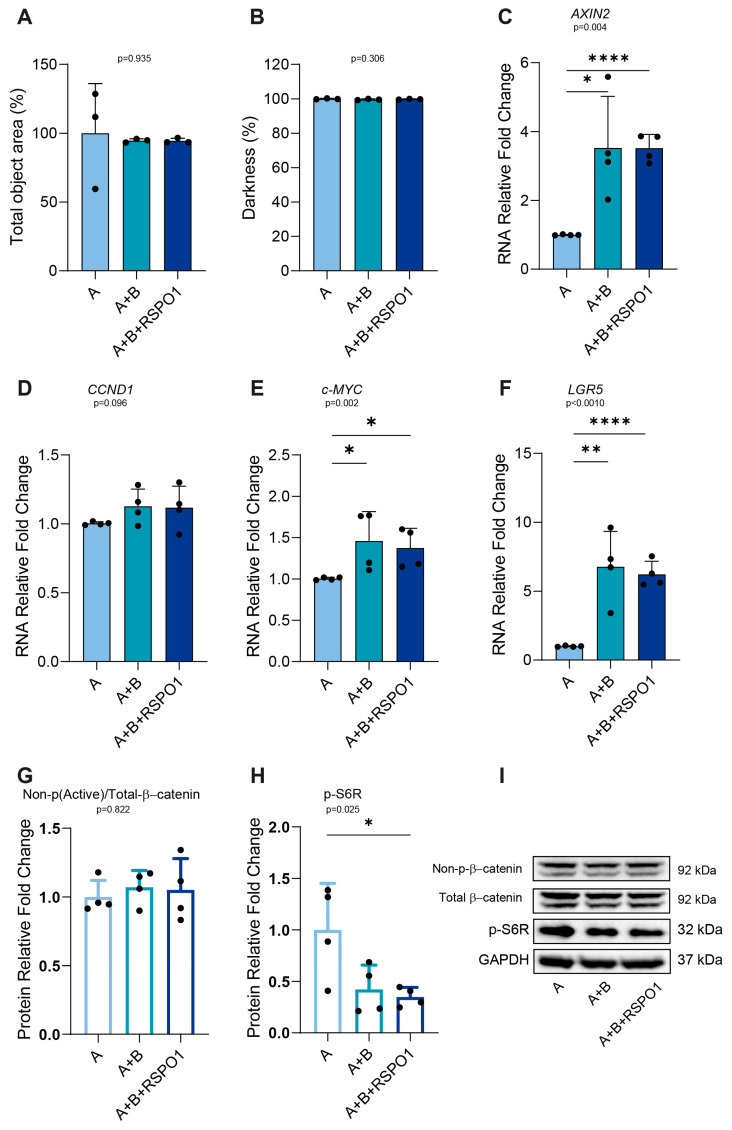
RSPO1 guides p-S6R downregulation in APC-mutated FAP NM PDOs independently from a boosted activation of LGR5. (**A**,**B**) Morphological analysis of the total object area and darkness of FAP NM PDOs cultured with the indicated media for 48 h (n = 3, FAP NM-03 = 3). (**C**–**F**) RNA expression analysis of the *AXIN2*, *CCND1*, *c-MYC* Wnt-target genes and *LGR5* intestinal marker in FAP NM PDOs cultured for 48 h with A, A+B, and A+B+RSPO1 (n = 4, FAP NM-03 = 4). (**G**,**H**) WB analysis of non-p(Active)/total β-catenin protein ratio and p-S6R in FAP NM PDOs cultured for 48 h with the indicated media (n = 4, FAP NM-03 = 4). (**I**) Representative WB imaging of non-p(Active)-β-catenin, total β-catenin, p-S6R, and GAPDH in FAP NM PDOs cultured for 48 h with the indicated media (n = 4, FAP NM-03 = 4); GAPDH was used as the loading control (cropped blots). Experiments were independently repeated at least three times and values are shown as the mean ± SD. Data were analyzed with the parametric Student’s *t*-test (morphological analysis, *LGR5*, non-p(Active)/total β-catenin and p-S6R), according to the Shapiro–Wilk normality test results. * *p* < 0.05, ** *p*  < 0.01, **** *p* < 0.0001. A = WNT3A-lacking commercial medium composed of DMEMF12 and Component A in a 1:1 ratio; A+B = WNT3A-containing commercial medium composed of Component A and Supplement B in a 1:1 ratio (exact formulation relative to other factors present is not known); A+B+RSPO1 = WNT3A-containing commercial medium composed of Component A and Supplement B in a 1:1 ratio, with RSPO1 3.

Once the genetic background and *LGR5* stemness marker as potential factors involved in the strength of the negative regulation of p-S6R were excluded, we decided to further assess whether RSPO1 fulfilled this role in the APC-mutated FAP NM PDO setting. To verify this hypothesis, we cultured FAP NM PDOs for 48 h with A, A+B, and A+B+RSPO1. Similar to CRC NM PDOs, we found that independently from the presence of WNT3A or RSPO1, none of the media used influenced the morphological features of the organoids or non-phospho (Active)/total-β-catenin protein expression (Figure 6A,B,G,I). More importantly, although the use of A+B or A+B+RSPO1 medium resulted in a significant increase in *AXIN2* (A+B, *p* = 0.014/A+B+RSPO1, *p* < 0.0001), *c-MYC* (A+B, *p* = 0.043/A+B+RSPO1, *p* = 0.022), and *LGR5* (A+B, *p* = 0.004/A+B+RSPO1, *p* < 0.0001) gene expression compared with the A medium (Figure 6C–F), a statistically significant reduction in p-S6R was exclusively observed upon the addition of RPSO1 (*p* = 0.030) (Figure 6H,I).

Notably, it was shown that a subgroup of PDOs derived from polyps and matched non-polyps from FAP patients, grown in media containing WNT and RSPO, presented no difference in *LGR5* mRNA expression [33]. This potentially supports the results that indicate the absence of an increase in *LGR5* transcriptional activation upon RSPO1 addition in our FAP NM PDOs. Our result further supports the idea that, in the presence of an APC-mutated intestinal setting, RSPO1 could directly guide p-S6R downregulation and that this regulation axis does not involve *LGR5* overexpression, as observed for the non-APC-mutated CRC NM PDOs. These data are also consistent with the recently proposed mechanism by which RSPO1 blocks the irradiation-induced phosphorylation of mTOR and its downstream targets (p-S6 and p-4EBP1) in bone mesenchymal stem cells [34].

## 3. Materials and Methods

### 3.1. Cell Culture

The HEK 293 STF cell line (CRL-3249™, ATCC) (Manassas, Virginia) was cultured in DMEMF12 (Biowest, #L0094500) (Bradenton, FL, USA) containing 20% heat-inactivated fetal bovine serum (FBS) (Microgem, #RM10342) (London, UK), 1% Glutamax (Gibco, #35050061) (Waltham, MA, USA), 1% penicillin/streptomycin (Gibco, #129211), and 200 µg/mL G418 (Gibco, #11811-023), also referred to in this paper as NC medium (+G418). The cell line was maintained under a humidified atmosphere with 5% CO_2_ at 37 °C in a cell incubator. Cells were validated through STR profiling. We periodically confirmed the absence of mycoplasma contamination.

### 3.2. Tissue Collection and Organoid Isolation

The study was approved by the ethical committee of the Sant’Orsola-Malpighi Hospital (EC: 599/2018/Sper/AOUBo, 2018/10/17) (Bologna, Italy). Informed consent was obtained from all human research participants. This study and experiments conformed to the principles set out in the WMA Declaration of Helsinki and the Department of Health and Human Services Belmont Report. Surgically resected intestinal tissues or endoscopic biopsy samples were obtained from consenting CRC and FAP patients, respectively, enrolled at the IRCCS Sant’Orsola-Malpighi Hospital (Bologna, Italy). In this study, sex and/or gender reporting was not relevant to the topic of our research. Morphologically appearing normal mucosa (NM) was collected from two recruited FAP (FAP NM-01 and 03) and three sporadic CRC (CRC NM 10, 13, and 16) patients. For CRC patients, NM was taken at a distance of more than 5 cm to the tumors. Absence or presence of APC mutations was confirmed by next generation sequencing (NGS) in CRC NM and FAP NM tissues, as previously reported [35]. Biopsy and tissue specimens were collected and cryopreserved, and patient-derived organoids were isolated according to the protocol by Tsai et al. with some modifications [36]. Briefly, for the CRC NM samples, the intestinal frozen tissue was quickly thawed in a water bath at 37 °C and washed once with D-PBS. Tissue fragments were cut into smaller fragments and digested with 200 U/mL collagenase type II (Gibco, #17101-015) in D-PBS for 1 h at 37 °C. The intestinal fragments were centrifuged (600× *g* for 5 min at 4 °C) and resuspended with a 1 mL syringe (21G needle) in DMEMF12 + 1% BSA. For the FAP NM samples, frozen biopsies were quickly thawed in water bath at 37 °C, and the freezing medium was replaced with cold recovery media (Commercial Component A + Supplement B, termed A+B, containing 2.5 µM of Thiazovivin, a ROCK inhibitor). Biopsies were cut into smaller fragments and centrifuged (290× *g* for 5 min, +4 °C). Gentle Cell Dissociation Reagent (GCDR) (Stem Cell Technologies, #100-0485) (Vancouver, Canada) was added, and the biopsy fragments were incubated at room temperature on a rocking platform for 20 min. The samples were then centrifuged (290× *g* for 5 min, +4 °C) and resuspended with a 1 mL syringe (21G needle) in DMEMF12 + 1% BSA. After digestion, the FAP NM and CRC NM samples were filtered through a 70 µm strainer, centrifuged, and resuspended in undiluted Matrigel^®^ (Corning, #356231) (Corning, NY, USA). Then, a 50 µL single Matrigel^®^ drop/well was seeded in a 24-well plate, and after solidification, 650 µL of A+B medium containing 2.5 µM of Thiazovivin (Thz) (Sigma-Aldrich #SML1045) (St. Louis, MO, USA) was added. Media were replaced every two days until the expansion procedures started.

### 3.3. APC Gene Target Sequencing

Genomic DNA was extracted from snap frozen FAP NM-01 and 03 biopsies using a Maxwell 16 instrument (Promega Corporation, Madison, WI, USA) and sequenced by Sanger sequencing. For each sample, the APC amplicon carrying the germline mutation was sequenced, based on the patients’ clinical reports. In detail, 30 ng of genomic DNA, extracted from fresh FAP NM biopsies, was amplified with AmpliTaq Gold DNA Polymerase (Life Technologies, Carlsbad, CA, USA) with a ProFlexTM 3 × 32-well PCR System (Thermo Fisher Scientific, Waltham, MA, USA). APC sequencing was performed on PCR products purified with the GeneJET PCR Purification Kit (Thermo Fisher Scientific). The primer sequences and annealing temperatures are reported below in Table 1.

### 3.4. Organoid Culture

For the expansion procedures, organoids were dissociated to single cells using TrypLE™ Express (Gibco, #12604-013), resuspended five times with a 1 mL syringe (21G needle), and incubated for 30 min at 37 °C, with vortexing every 5 min. The reaction was stopped with FBS. The organoids were dissociated and resuspended again (20 times) with a 1 mL syringe (21G needle) into a single cell suspension. Cells were centrifuged at 500× *g* for 3 min and counted. A total of 15,000 cells/well (50 µL drop/well) were seeded in a 24-well plate in A+B medium with 2.5 µM Thz. Organoids were passaged every 5–7 days. When intermediate dimensions (around 100 µm/organoids) were reached, the organoids were gently dissociated with GCDR and seeded in a 24-well plate for protein and RNA extraction or reduced to single cells using TrypLE™ Express (Thermo Fisher Scientific) and seeded in a 96-well plate for the viability assay. For 24-well plate seeding, organoids were dissociated in GCDR for 10 min at RT with constant agitation. After centrifugation (290× *g* for 5 min), the organoids were resuspended with a 1 mL syringe (21G needle) 10 times in cold DMEMF12 + 1% BSA. Organoids were filtered in a 70 µm strainer, centrifuged again, and seeded (1:4) in a 24-well plate (50 µL drop/well). For the viability assay, the organoids were dissociated into single cells with TrypLE™ Express, as described above. Cells (10,000 cells/well) were seeded in a 96-well plate (25 µL drop/well). After the expansion procedures, the medium was always supplemented with Thz. According to the type of experiment, the organoids were cultured in different growth media formulations, as detailed in Table S1. Notably, the exact formulation of the commercial A (Component A + DMEMF12 in 1:1 ratio) and A+B (Component A + Supplement B in a 1:1 ratio) medium is not known, and the only related information is that the A medium is used to establish and grow Wnt-independent intestinal tumor organoids, while the A+B medium is indicated for the establishment and long-term maintenance of normal intestinal organoids derived from human intestinal crypts. As a result, A and A+B media were reported in this work as WNT3A-lacking or WNT3A-containing commercial media, respectively. After the indicated treatment times, the organoids were harvested with Cultrex Harvesting Solution (Biotechne, #3700-100-01) (Minneapolis, MN, USA) according to the manufacturer’s instructions, and pellets were collected for further biochemical analysis. All of the experiments were conducted between passages two and three.

### 3.5. Organoid Viability

For the organoid viability experiments, the organoids were dissociated into single cells with TrypLE™ Express and seeded as described above. After the treatments (48 h), the organoid viability was analyzed by the CellTiter-Glo^®^ 3D Cell Viability Assay (Promega, #G9681) according to the manufacturer’s instructions. The luminescent signal was measured by the multimode plate reader Spark^®^ (Tecan) (Männedorf, Switzerland) (integration time 1000 ms). Reactions were performed in triplicate.

### 3.6. Imaging

The organoids’ morphological changes (total object area and darkness) were monitored every 8 h for 48 h through the Incucyte^®^ Live Cell Analysis System (Sartorius) (Göttingen, Germany) and analyzed by the Incucyte^®^ Organoid Software Module 2022B Rev2 GUI (Sartorius, #9600-0034). The organoids’ total object area, object count, and darkness were important morphological characteristics chosen as the main indicators of the growth/proliferation status and collapse of the organoids. We measured the darkness metric as the number of dark organoid objects in the area selected inside the well calculated on the object average in the same area; the total area of organoids is the area occupied by organoids in the area selected inside the well calculated on the object average in the same area; and the organoid count is the ratio between the number of organoids and the area occupied by organoids in each image. T0 was used for the normalization of the metric measurements. For a single experiment, each analysis was performed on four different images for each different media. Representative imaging was performed through Incucyte^®^ and an EVOS M5000 microscope (Invitrogen™; Thermo Fisher Scientific, Waltham, MA, USA).

### 3.7. Luciferase Assay

For the cell-based luciferase assay, HEK 293 STF cells were used as reported by Pleguezuelos-Manzano et al. [37]. Cells (36,000 cells/well) were cultured with the media indicated in Table S1. NC was used as the negative control while PC1 and PC2, containing 1 µM or 10 µM of CHIR99021 (positive Wnt activator), respectively, were used as the positive controls. Luciferase activities were measured by the Dual-Luciferase^®^ Reporter Assay System (Promega, #E1910) according to the manufacturer’s instructions. Reactions were performed in quadruplicate.

### 3.8. RNA Extraction and Quantitative PCR

The total RNA was extracted from the organoid pellets using the TRIzol^®^ reagent (Invitrogen™; Thermo Fisher Scientific, #15596018) according to the manufacturer’s instructions. RNA quantification and purity evaluation were performed by using a NanoDrop 1000 Spectrophotometer (Thermo Fisher Scientific). A total of 2 µg of RNA extracted from the organoids was reverse transcribed using the High-Capacity cDNA Reverse Transcription Kit (Applied Biosystems™, Thermo Fisher Scientific, #4374966) according to the manufacturer’s protocol. Quantitative real-time PCR (qPCR) was performed on a QuantStudio5 thermal cycler (Applied Biosystems™; Thermo Fisher Scientific) using the TaqMan Fast Advanced Master Mix (Applied Biosystems™; Thermo Fisher Scientific, #4444557). The mRNA expression was analyzed using the following TaqMan probes: *AXIN2* (Assay ID: Hs00610344_m1), *c-MYC* (Assay ID: Hs00153408_m1), *CCND1* (Assay ID: Hs00765553_m1), *LGR5* (Assay ID: Hs00969422_m1), *KRT20* (Assay ID: Hs00300643_m1), and *GAPDH* (Assay ID: Hs03929097_g1). Fold change values were calculated using the 2^−ΔΔCt^ method. *GAPDH* was used as the reference gene for the normalization of qPCR data. Reactions were performed in triplicate.

### 3.9. Western Blot Analysis

The total proteins were extracted by using RIPA Buffer (made of Tris-HCL Ph-7.5 25 mM, NaCl 150 mM, Na-Deoxycholate 0.50%, NP-40 1%, SDS 0.10% and water) (Thermo Fisher Scientific). A total of 15 µg of total proteins/sample were separated on 4–12% NuPAGE™ Novex Bis-Tris gels (Invitrogen™, Thermo Fisher Scientific, #NP0335BOX) using the NuPAGE™ MOPS SDS Running Buffer (Invitrogen™, Thermo Fisher Scientific, #NP0001). Then, proteins were transferred onto a nitrocellulose membrane. Membranes were blocked with 5% non-fat milk diluted in PBS with 0.05% Tween^®^ 20 (PBST) and then incubated overnight at +4 °C with the following primary antibodies: non-phospho (Active) β-catenin (1:2000, CST #8814), total β-catenin (1:2000, CST #9562), phospho-S6 ribosomal protein (p-S6R) (Ser235/236) (1:2000, CST #2211), survivin (1:1000, CST #2808), and GAPDH (1:6000, CST #2118). GAPDH was used as the loading control.

After incubation with the primary antibody, membranes were incubated with the appropriate secondary antibody (Amersham, #NA934) for 1 h at room temperature. Secondary antibodies were diluted 1:1000 in 2% dried non-fat milk diluted in PBST.

Prior to re-probing with different antibodies, the membranes were stripped with Restore^TM^ Western Blot Stripping Buffer (Thermo Fisher Scientific, #21059), washed in PBST, and blocked in 5% non-fat milk diluted in PBST.

Signals were detected with Westar Eta C Ultra 2.0 (Cyanagen, #XLS075) (Bologna, Italy) and Westar Antares (Cyanagen, #XLS0142) chemiluminescent substrates. Images were acquired with a Chemidoc^TM^ XRS+ (Bio-Rad Laboratories) (Hercules, CA, USA). Densitometric analyses were performed with Image-Lab software, version 6.1 (Bio-Rad) to quantify the fold differences in protein expression. Uncropped blots and Red Ponceau relative to the corresponding main figures are reported in the Supplementary File.

### 3.10. Statistical Analysis

Statistical analysis was performed using GraphPad Software 8 (Graph Pad Software Inc., La Jolla, CA, USA). Data were analyzed with the parametric Student’s unpaired *t*-test or non-parametric Mann–Whitney test according to the results of the Shapiro–Wilk normality test. Global *p*-value calculation was performed using ordinary One-way ANOVA or Kruskal–Wallis test according to the results of the Shapiro–Wilk normality test. For the luciferase-based assay, NC was used as the reference media. For the WNT3A-CM quality test, the data were first normalized to the NC for each time point and then to NC t0. For the CRC NM PDO experiments, A and BASAL were chosen as the references for the commercial and home-made media, respectively. For the CRC NM and FAP NM comparative experiments, the corresponding A medium was used as the reference for each experimental group. For the FAP NM PDO experiments, A-FAP NM was used as the reference condition. Statistical significance was assessed as * *p* < 0.05, ** *p* < 0.01, *** *p* < 0.001, and **** *p* < 0.0001.

## 4. Conclusions

In this study, for the first time, we unveiled the additional impact of WNT3A-containing home-made media on the crosstalk between the Wnt/β-catenin (Wnt) and PI3K/mTOR (mTOR) pathways over the commercial one, proposing RSPO1 as a key regulatory factor. Particularly, we showed that the Wnt-enhancer-RSPO1 guides a negative regulatory axis between Wnt and mTOR, downregulating the mTOR downstream effector p-S6R in both the APC and non-APC mutated intestinal patient-derived organoids (PDOs). Interestingly, we highlight that the RSPO1–p-S6R axis was characterized by a transcriptional *LGR5* boosting only in the non-APC mutated CRC NM PDOs. To the best of our knowledge, this is the first attempt aiming to compare the effect of the use of commercial and home-made media on the interplay between Wnt and mTOR, eliciting the leading role of RSPO1 acting on a negative regulation between the two pathways. Our data could represent a methodological guide that could be extremely useful for scientists working in these fields to select the most appropriate intestinal patient-derived organoid growth media, depending on the purpose and/or experimental design. Indeed, in the study of cross-talk between the molecular pathways involved in CRC onset and progression or therapeutical resistance, the use of commercial over home-made media containing RSPO1 should be preferred as it did not exacerbate the negative feedback involving Wnt and mTOR.

In addition, due to its suppressive role toward p-S6R in both the APC and non-APC mutated setting, the inclusion of RSPO1 in the culture medium for intestinal PDOs needs to be carefully evaluated, especially in preclinical studies assessing the efficacy of Wnt or mTOR inhibitors or investigating the interplay between mTOR and the other molecular pathways underlying CRC carcinogenesis. Moreover, since the short-term treatments (48 h) with commercial or home-made WNT3A-lacking/containing media influenced neither the organoid viability nor morphological features, their use is interchangeable in the drug screening assay, except for compounds acting against markers involved in the Wnt or mTOR pathways. This methodological choice should also take into account that while the ready-to-use commercial media ensure reproducibility and stability, they are pricey, omit the exact formulation and/or the concentration of each component that might influence the activation status of the Wnt and mTOR pathways, and they cannot be modified according to the mutational setting of PDOs, except for those involving Wnt derangement. Our results showed that even at 88 h, there were no significant differences in the organoid viability, proliferation, and morphology in the CRC NM PDOs cultured with WNT3A-lacking/containing commercial or home-made media, supporting the interchangeability of commercial or home-made media, above all, in the drug screening assay. However, one limitation of this study is that we did not investigate the molecular changes in the Wnt and mTOR pathways in the long-term culture condition and after multiple passaging of the same organoids. This set of analyses was not conducted because of (i) the lack of available material to delve into the molecular perturbations of the two pathways at each passaging, and because (ii) we preferred to focus more on the experiments set between passages two and three, as it has been described that organoids at late passages (after passage four) and after 3 months in culture conditions could acquire new genetic or epigenetic mutations as well as phenotypic changes with morphological transition, reduced numbers of Lgr5+ stem cells, and the inconsistent expression of markers for differentiated intestinal epithelial cell types, which would have led to natural or confounding results regarding the activation of the two pathways [38,39]. Otherwise, home-made media are cost-effective and component-detailed, but the lack of standard shared formulations may affect the reproducibility. This study has further limitations due to the possible batch-to-batch variation of the conditioned media and Matrigel^®^, which may have decreased the strength of our data. In order to overcome these limitations, we increased the number of experiments and included PDOs derived from additional patients. In addition, we did not analyze the activation levels of the LGR5 protein. We are aware that RSPO1 binds to the LGR5 receptor, activating Wnt signaling through a positive feedback loop. However, the main goal of our methodological guide is to provide a comparative molecular analysis of the differential impact of WNT3A and RSPO1 in home-made and commercial intestinal PDO media on the Wnt and mTOR crosstalk. Accordingly, we mainly focused our attention on the activation of Wnt target genes including LGR5 and the mTOR downstream effector p-S6R.

## Figures and Tables

**Table 1:** Sequences of the APC exon primers with the relative length and annealing temperature (A.T.). Reference genome GRCh37/hg19.

FAP Patient	Amplicon	Forward Primer (5′ → 3′)	Reverse Primer (5′ → 3′)	Size (bp)	A.T.
FAP NM 01	Exon 16	TGGAGAACTAGATACACCAATA	CGTTCACTATAATTGGTAGGC	384	56 °C
FAP NM 03	Exon 9	CAGACACTTCATTTGGAGTACC	AGTAGAGATGGGGTTTTGCC	369	60 °C

## Data Availability

All data generated or analyzed during this study are included in this published article and its Supplementary Materials. Additional information can be requested from the corresponding author.

## Supplementary Materials

The following supporting information can be downloaded at: https://www.mdpi.com/article/10.3390/ijms252111526/s1.
