# Supplementary material for: Comparative Analysis of Commercial and Home-Made Media on RSPO1/S6R Axis in Organoids with Different Wnt Backgrounds: A Methodological Guide for the Selection of Intestinal Patient-Derived Organoids Culture Media
Source: Int J Mol Sci. 2024 Oct 26;25(21):11526. doi: 10.3390/ijms252111526 (PMC11546270; doi:10.3390/ijms252111526)
Supplement: Supplementary file 1 [file ijms-25-11526-s001.zip › ijms-3223685-supplementary.pdf]

Supplementary Materials

Table S1. Composition of media.

NC			
**Components**	**Final concentration**	**Company**	**Product number**
DMEMF12	Up to volume	Biowest	L0094500
FBS	20%	Microgem	RM10342
GLUTAMAX	1%	Gibco	35050061
Penicillin/Streptomycin	1%	Gibco	15140122

PC1			
**Components**	**Final concentration**	**Company**	**Product number**
NC	Up to volume		
CHIR99021	1 µM	Sigma	SML1046

PC2			
**Components**	**Final concentration**	**Company**	**Product number**
NC	Up to volume		
CHIR99021	10 µM	Sigma	SML1046

Commercial media				
**Components**	**Final concentration**		**Company**	**Product number**
	**A**	**A+B**		
DMEMF12	50%	/	Biowest	L0094500
Component A	50%	50%		
Supplement B	/	50%		
Penicillin/Streptomycin	1X	1X	Gibco	15140122

BASAL			
**Components**	**Final concentration**	**Company**	**Product number**
Advanced DMEM-F12	Up to volume	Gibco	#12634-010
HEPES	1X	Gibco	#15630080
Glutamax	1X	Gibco	#35050061
Penicillin/Streptomycin	1X	Gibco	#129211
N2	1X	Gibco	#17502-048
B27	1X	Gibco	#17504-044
N-Acetylcysteine	1 nM	Sigma-Aldrich	#A7250
Gastrin	10 nM	Sigma-Aldrich	#G9145
A83-01	500 nM	Tocris	#2939
SB202190	10 µM	Sigma-Aldrich	#S7067
Nicotinamide	10 mM	Sigma-Aldrich	#N0636
mEGF	50 ng/ml	Gibco	#PMG8041
Human-Noggin	100 ng/ml	Peprotech	#120-10C

+/-WNT3A-CM; +/-RSPO1			
**Components**	**Final concentration**	**Company**	**Product number**
BASAL	50%		
WNT3A-conditioned medium (CM)	50%	[37]	
Human Protein RSPO1	1 µg/ml	Biotechne	#4645-RS

Table S2. APC mutations in the normal mucosa of FAP patients.

FAP Patient	Coding region	Protein	Function
FAP NM 01	c.3202-3205delTCAA	p.Ser1068fs	Frameshift
FAP NM 03	c.847C>T	p.Arg283Ter	Nonsense

Figure S1. Quality control test of WNT3A-CM over time.

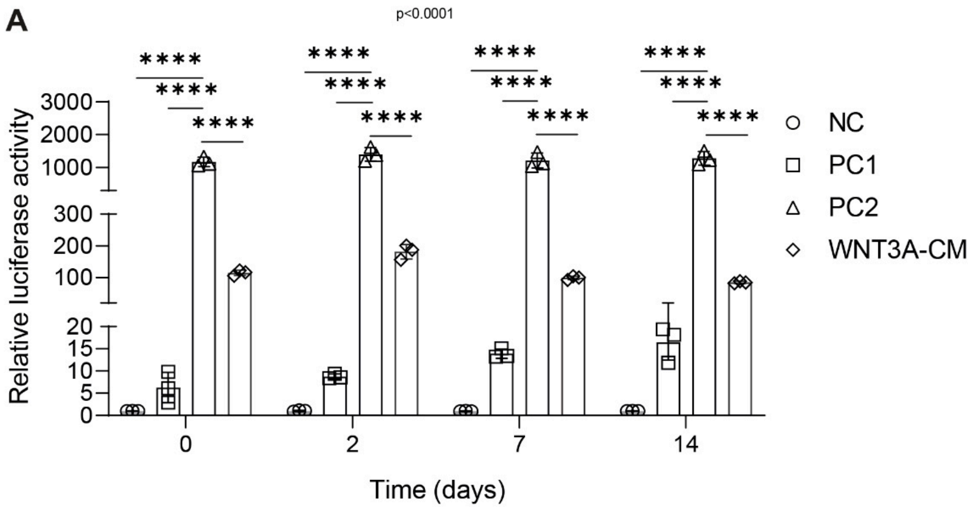

Figure S1. Quality control test of WNT3A-CM over time.
(A) Luciferase-based quality control test of WNT3A-CM in HEK 293 STF cells cultured for 24 h with freshly thawed medium (termed 0) or 2/7/14-days thawed medium (n = 3); NC and PC1/2 were used as the negative and positive controls, respectively.
Experiments were independently repeated three times and the values are reported as the mean ± SD. Data were analyzed with the parametric Student's t-test, according to the Shapiro–Wilk normality test results. **** p <0.0001.

Figure S2. Short-term and long-term representative imaging of CRC NM PDOs cultured with commercial and home-made media.

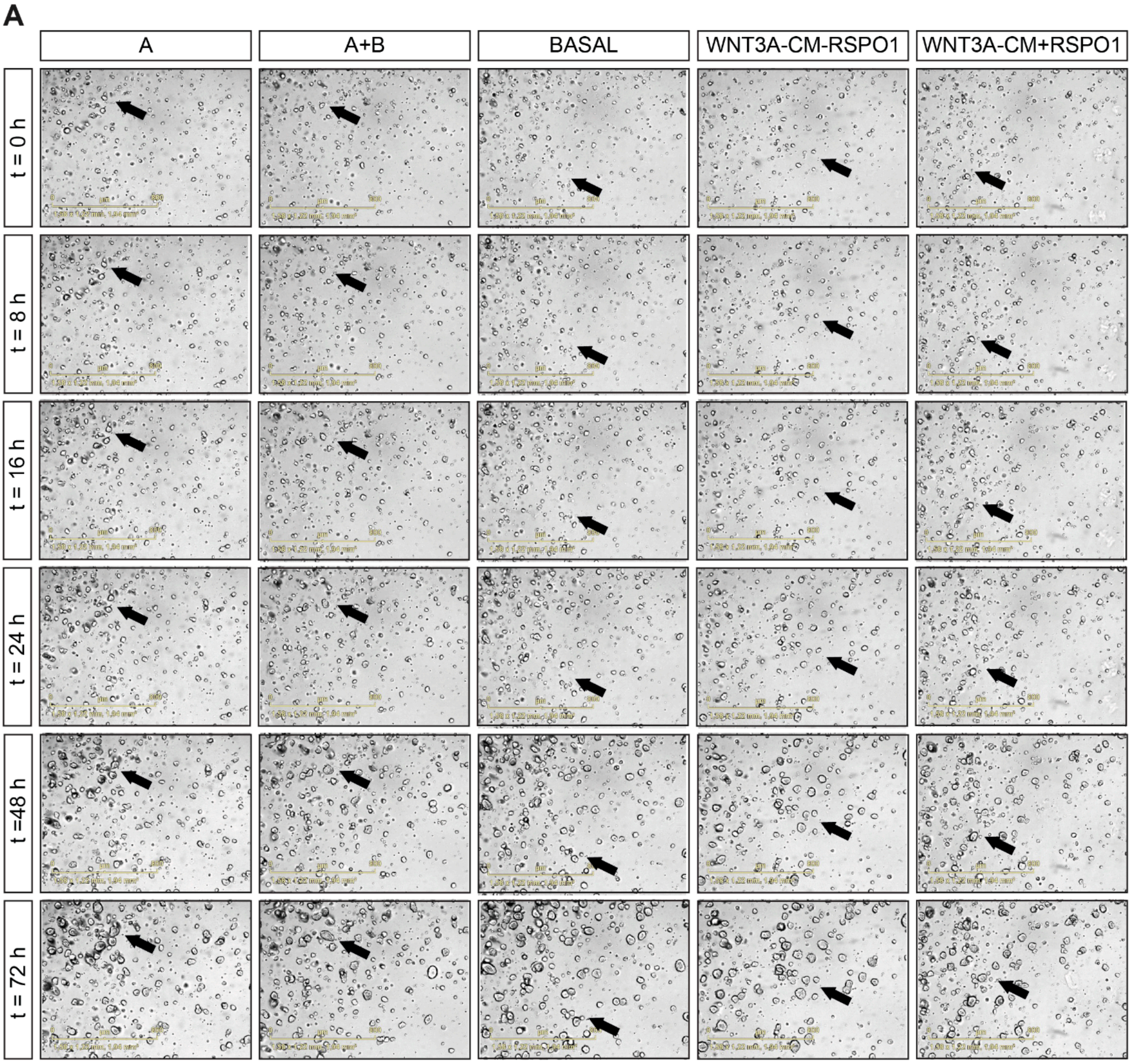

Figure S2. Short-term and long-term effects of commercial and home-made media on the CRC NM PDO morphology. (**A**) Representative images of CRC NM PDOs cultured with the indicated media over time. Representative imaging was performed through Incucyte software. A = WNT3A-lacking commercial medium composed of DMEMF12 and Component A in a 1:1 ratio; A+B = WNT3A-containing commercial medium composed of Component A and Supplement B in a 1:1 ratio (exact formulation relative to other factors present is not known); BASAL = WNT3A-lacking home-made basal medium; WNT3A-CM = WNT3A-containing complete home-made medium, with or without R-spondin 1 (+/-RSPO1). Black arrow points to the growth of the same organoid or group of organoids over time.

Figure S3. Short-term and long-term morphological analysis of CRC NM PDOs cultured with commercial and home-made media.

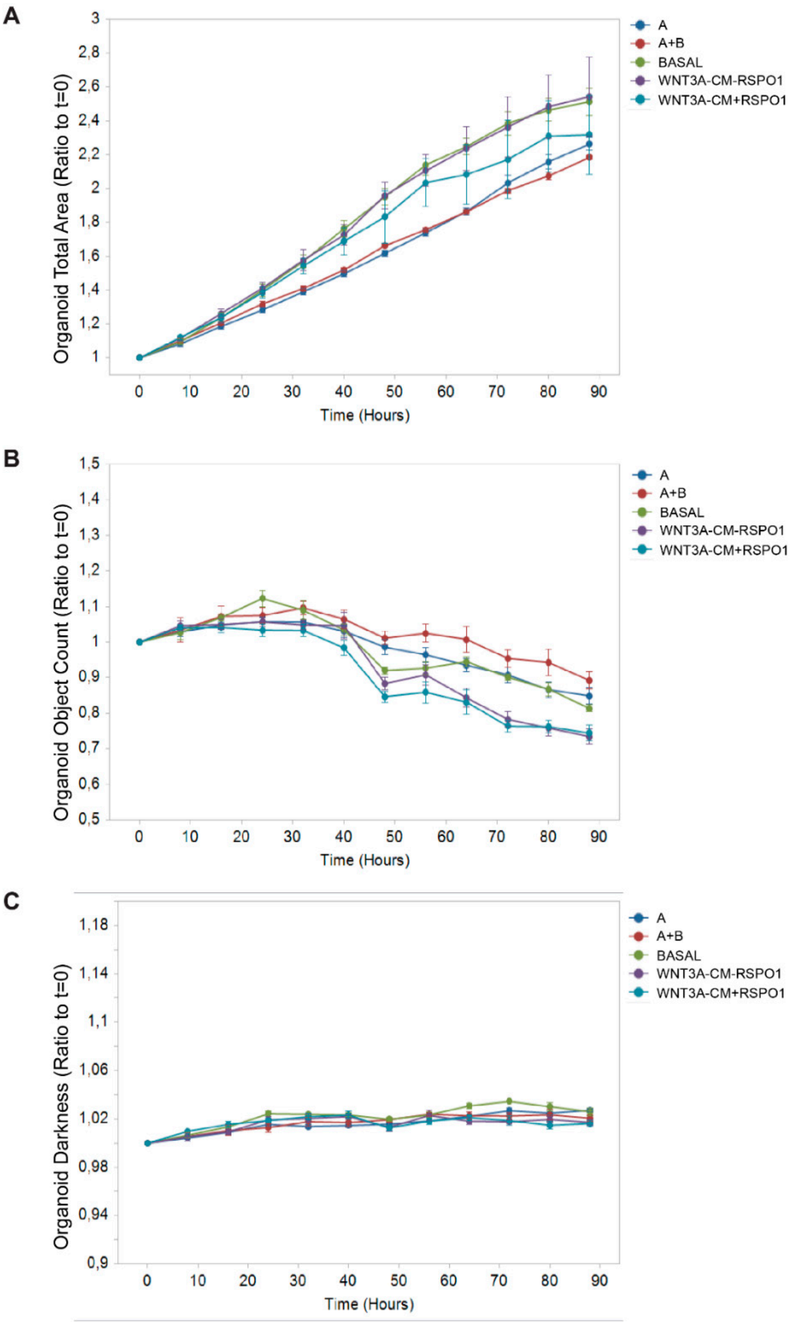

Figure S3. (A–C) Morphological analysis of the organoids' total object area, object count, and darkness of the CRC NM PDOs cultured with the indicated media over time. Changes in the organoids' total object area, object count, and darkness were monitored every 8 h for 88 h and analyzed through Incucyte software. A = WNT3A-lacking commercial medium composed of DMEMF12 and Component A in a 1:1 ratio; A+B = WNT3A-containing commercial medium composed of Component A and Supplement B in a 1:1 ratio (exact formulation relative to other factors present is not known); BASAL = WNT3A-lacking home-made basal medium; WNT3A-CM = WNT3A-containing complete home-made medium, with or without R-spondin 1 (+/-RSPO1).

Supplementary File
Uncropped blots relative to Main Figures 1, 2, and 3.

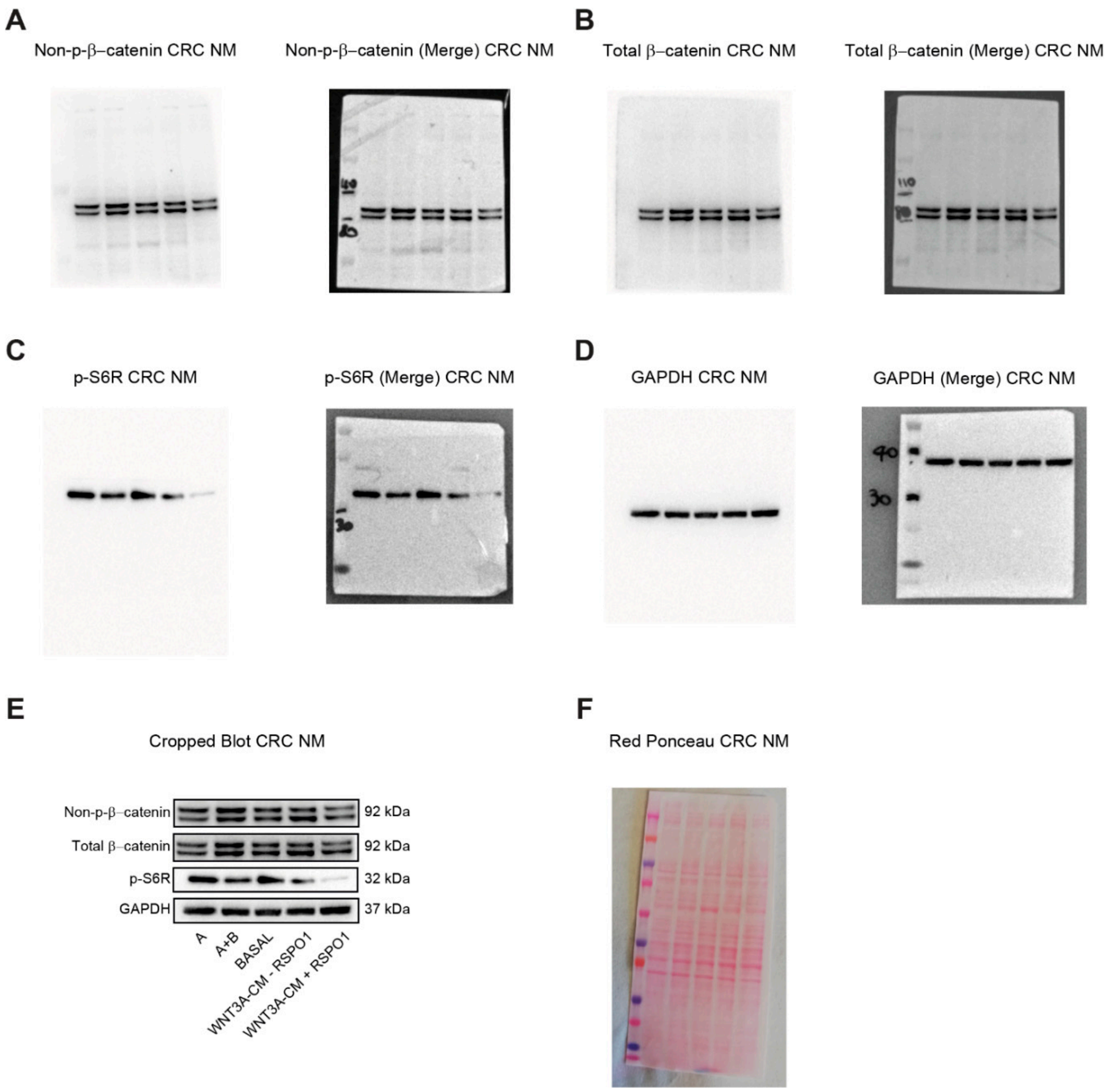

Uncropped blots relative to Main Figures 1, 2, and 3. Representative uncropped blots of non-p(Active)-β-catenin, total β-catenin, p-S6R, and GAPDH (n=7) in the CRC NM PDOs cultured for 48 h with the indicated media.

(A) Uncropped blots of non-p(Active)-β-catenin (original, left; merged with the Protein Standard, right).

(B) Uncropped blots of total β-catenin (original, left; merged with the Protein Standard, right).

(C) Uncropped blots of p-S6R (original, left; merged with the Protein Standard, right).

(D) Uncropped blots of GAPDH (original, left; merged with the Protein Standard, right).

(E) Cropped blots relative to Main Figure 1.

(F) Red Ponceau of the transferred proteins before being divided into two parts to stain proteins ranging from 260 kDa to 60 kDa in the upper part or from 50 kDa to 3.5 kDa in the lower part.

Uncropped blots relative to Main Figure 4.

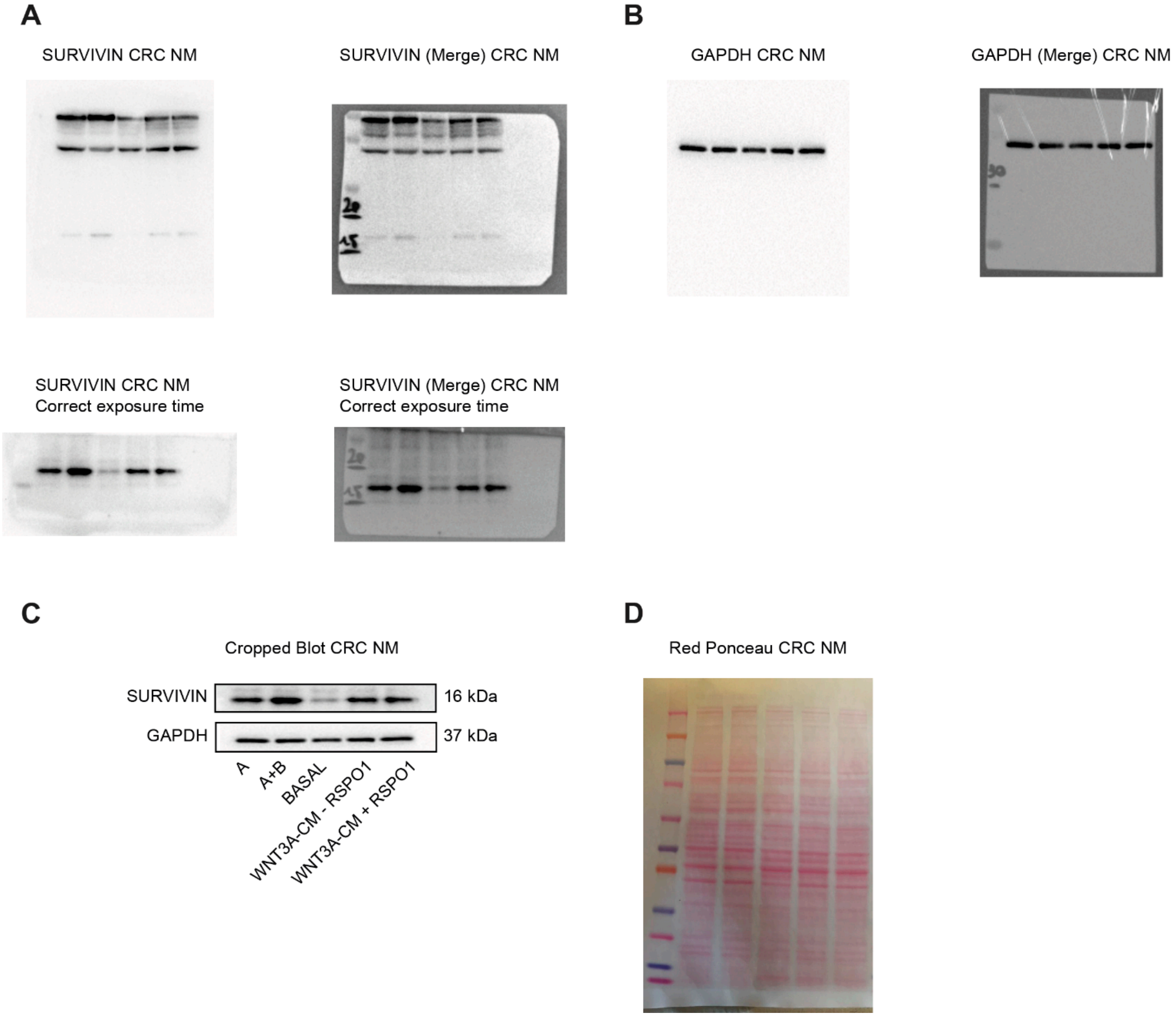

Uncropped blots relative to Main Figure 4. Representative uncropped blots SURVIVIN and GAPDH (n = 6) in the CRC NM PDOs cultured for 48 h with the indicated media. For SURVIVIN signal acquisition, due to the high signal belonging to the higher molecular weight proteins tested, we covered the upper part of the blot to acquire the SURVIVIN protein signal with the optimized exposure time already selected in our lab for all of the experiments.

(A) Uncropped blots of SURVIVIN (original, upper left; merged with the Protein Standard, upper right; original covered, bottom left; merged with the Protein Standard covered, bottom right).

(B) Uncropped blots of GAPDH (original, left; merged with Protein Standard, right).

(C) Cropped blots relative to Main Figure 2.

(D) Red Ponceau of the transferred proteins before being divided in two parts to stain proteins ranging from 260 kDa to 60 kDa in the upper part or from 50 kDa to 3.5 kDa in the lower part.

Uncropped blots relative to Main Figure 5.

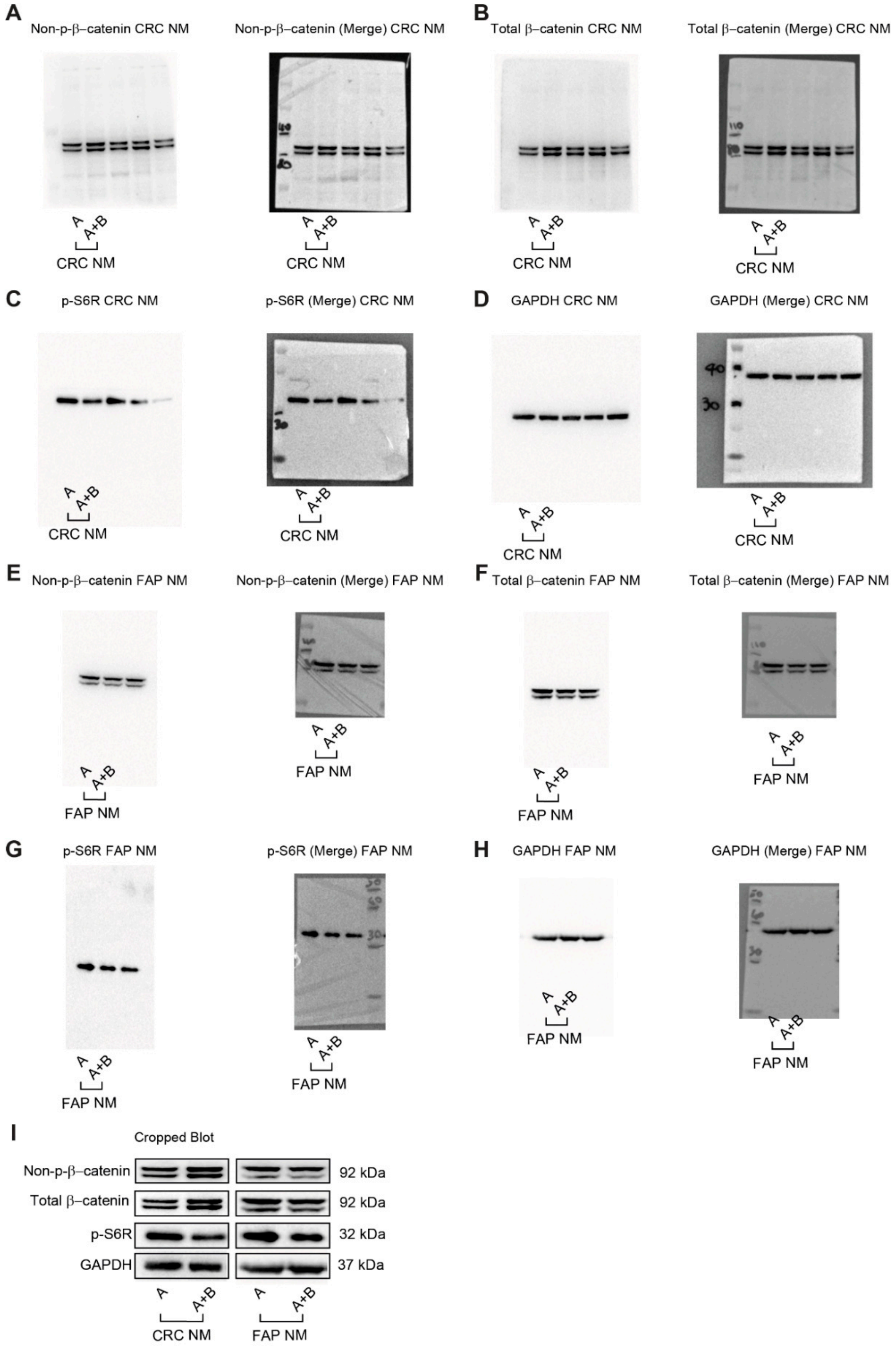

Uncropped blots relative to Main Figure 5. Representative uncropped blots of non-p(Active)-β-catenin, total β-catenin, p-S6R, and GAPDH (n = 5) in the CRC NM and FAP NM PDOs cultured for 48 h with the indicated media. The representative WB images of CRC NM (A/A+B) and FAP NM (A/A+B) were selected to better recapitulate the mean values shown in the corresponding histograms. For this reason, the representative imaging selected for CRC NM (A/A+B) and FAP NM (A/A+B) belongs to different blots that included other conditions together with the experimental conditions of interest in the presented study. Due to the low concentration of proteins extracted from the intestinal PDOs, we could not reload the blot again following the desired order of samples.

(A) Uncropped blots of non-p(Active)-β-catenin in the CRC NM PDOs (original, left; merged with the Protein Standard, right).

(B) Uncropped blots of total β-catenin in the CRC NM PDOs (original, left; merged with the Protein Standard, right).

(C) Uncropped blots of p-S6R in the CRC NM PDOs (original, left; merged with the Protein Standard, right).

(D) Uncropped blots of GAPDH in the CRC NM PDOs (original, left; merged with the Protein Standard, right).

(E) Uncropped blots of non-p(Active)-β-catenin in the FAP NM PDOs (original, left; merged with the Protein Standard, right).

(F) Uncropped blots of total β-catenin in the FAP NM PDOs (original, left; merged with the Protein Standard, right).

(G) Uncropped blots of p-S6R in the FAP NM PDOs (original, left; merged with the Protein Standard, right).

(H) Uncropped blots of GAPDH in the FAP NM PDOs (original, left; merged with the Protein Standard, right).

(I) Cropped blots relative to Main Figure 3.

Uncropped blots relative to Main Figure 6.

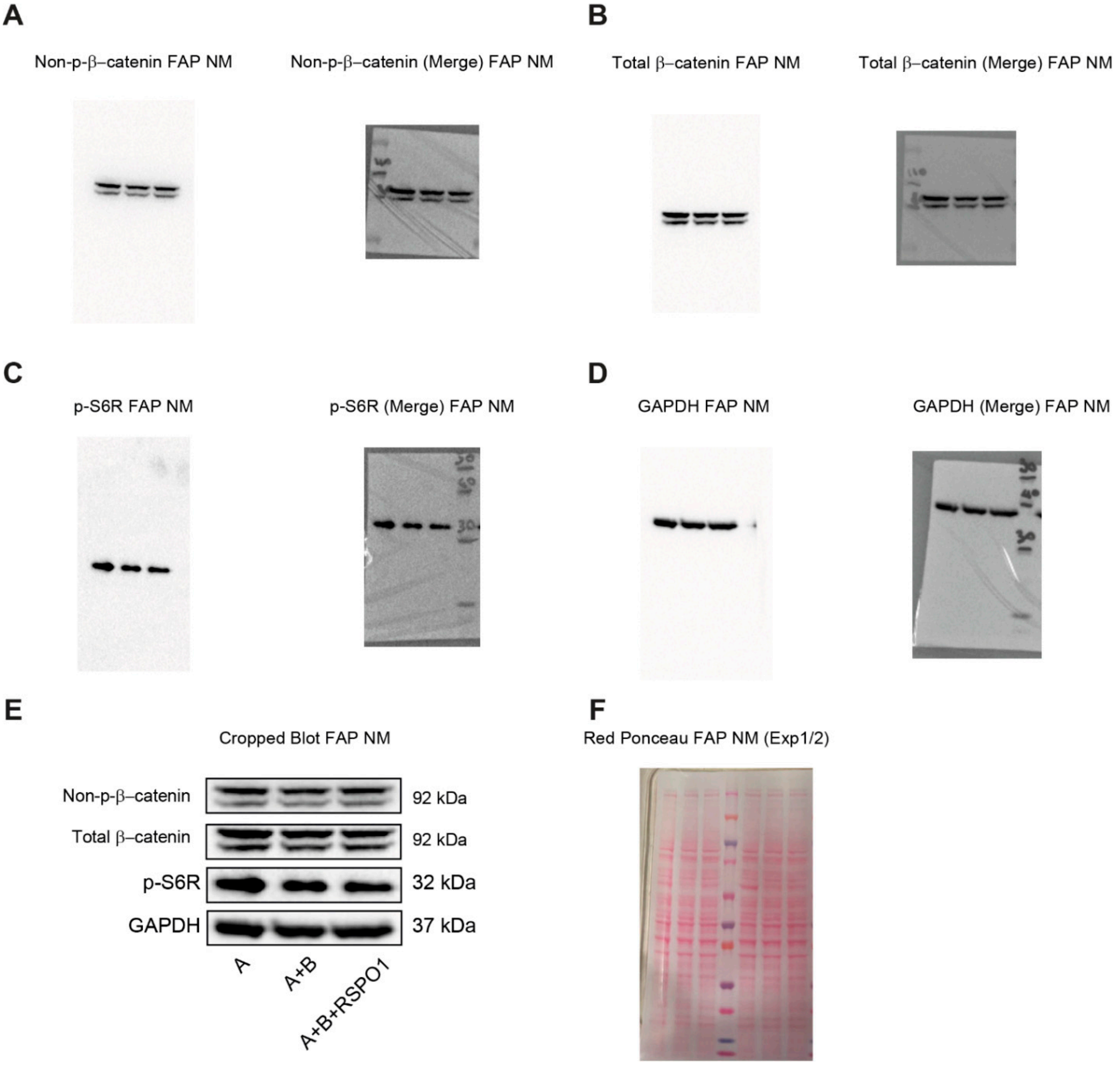

Uncropped blots relative to Main Figure 6. Representative uncropped blots of non-p(Active)-β-catenin, total β-catenin, p-S6R, and GAPDH (n = 4) in the FAP NM PDOs cultured for 48 h with the indicated media.

(A) Uncropped blots of non-p(Active)-β-catenin (original, left; merged with the Protein Standard, right).

(B) Uncropped blots of total β-catenin (original, left; merged with the Protein Standard, right).

(C) Uncropped blots of p-S6R (original, left; merged with the Protein Standard, right).

(D) Uncropped blots of GAPDH (original, left; merged with the Protein Standard, right).

(E) Cropped blots relative to Main Figure 4.

(F) Red Ponceau of the transferred proteins before being divided into two parts to stain proteins ranging from 260 kDa to 60 kDa in the upper part or from 50 kDa to 3.5 kDa in the lower part.
